# Cross-Cultural Validity Study of a Medical Education Leadership Competencies Instrument in Latin American Physicians: A Multinational Study

**DOI:** 10.1200/JGO.19.00243

**Published:** 2019-11-26

**Authors:** Max S. Mano, Rafaela Gomes, Gustavo Werutsky, Carlos H. Barrios, Gustavo Nader Marta, Cynthia Villarreal-Garza, Antonio Luiz Frasson, Cinthya Sternberg, Renan Clara, Sergio D. Simon, Fadil Çitaku, Marianne Waldrop, Claudio Violato, Don Zillioux, Yawar Hayat Khan

**Affiliations:** ^1^ Latin American Cooperative Oncology Group, Porto Alegre, Brazil; ^2^ Hospital Sírio-Libanês, São Paulo, Brazil; ^3^ Instituto do Câncer do Estado de São Paulo of the Universidade de São Paulo, Sao Paulo, Brazil; ^4^ Hospital Zambrano Hellion-Tecnológico de Monterrey, San Pedro Garza García, Mexico; ^5^ Instituto Nacional de Cancerologia, Ciudad de México, México; ^6^ Brazilian Society of Mastology, Sao Paulo, Brazil; ^7^ Brazilian Society of Clinical Oncology, Sao Paulo, Brazil; ^8^ Academy of Leadership Sciences Switzerland, Zürich, Switzerland

## Abstract

**PURPOSE:**

Physicians rarely receive formal training in leadership skills. Çitaku and colleagues have identified a set of leadership competencies (LCs) providing validity evidence in North American (NA) and European Union (EU) medical education institutions. We aim to apply this same survey to a sample of Latin American (LA) medical leaders from the oncology community and related areas, compare the results with those of the previous survey, and perform subgroup analyses within the LA cohort.

**METHODS:**

The survey was sent to nearly 8,000 physicians of participating professional organizations. In addition to the 63 questions, we also collected data on the type of institution, country, specialty, sex, age, years of experience in oncology, and leadership position.

**RESULTS:**

The 217 LA respondents placed the highest value on task management competencies (91.37% reported these as important or very important *v* 87.0% of NA/EU respondents; *P* < .0001), followed by self-management (87.45% of LA respondents *v* 87.55% of NA/EU respondents; *P* = not significant [NS]), social responsibility (86.83% of LA respondents *v* 87.48% of NA/EU respondents; *P* = NS), innovation (86.69% of LA respondents *v* 85.31% of NA/EU respondents; *P* = NS), and leading others (83.31% of LA respondents *v* 84.71% of NA/EU respondents; *P* = NS). Social responsibility, which was first in importance in the NA/EU survey, was only third in the LA survey. Subgroup analyses showed significant variations in the ratings of specific LCs within the LA population.

**CONCLUSION:**

LCs valued by LA leaders somewhat differ from those valued by their NA and EU counterparts, implying that cultural aspects might influence the perception of desired LCs. We also detected variations in the responses within the LA population. Our data indicate that current physician leadership training programs should be tailored to suit specific needs and cultural aspects of each region. Further validity studies of this instrument with other samples and cultures are warranted.

## INTRODUCTION

Leadership has been pragmatically defined as “a process of motivating people to work together collaboratively to accomplish great things.”^1(p18)^ Effective leadership has become critical for health care institutions as they face growing challenges with competition, regulatory complexity, job hopping of qualified personel,^2^ and escalating health care costs,^3^ only to mention a few. In the medical field, effective leadership requires the development of a set of competencies; a successful career as a physician, educator, or researcher is no guarantee of success in a leadership position.^4,5^ Despite this, training physicians in leadership skills has not been routine practice in health care institutions.^6^

Numerous studies have demonstrated the benefits of teaching leadership in medical schools.^7,8^ As expected, the availability of physician leadership training programs has increased,^9^ as has the number of scientific publications addressing this subject.^10^ Recently, the effectiveness of these training programs has also been scrutinized.^11^ In Latin America (LA), which is the target region of this study, medical leadership has been poorly addressed, despite the pressing need for well-trained medical leaders in the region.^3^

The acclaimed Global Leadership and Organizational Behavior Effectiveness (GLOBE) survey, performed in the business realm, demonstrated that required or desired leadership competencies (LCs) have wide variations according to a number of factors, such as sex, age, years of experience in a leadership position, area of expertise, and cultural background.^12^ Studies performed in the medical field reported similar conclusions; in a large Turkish survey focused on family physicians, emotional intelligence scores varied significantly according to sex, geographic region, and experience, and the emotional intelligence scores correlated significantly with leadership traits.^13^

**CONTEXT SUMMARY**

**Key Objective**
Leadership competencies are not routinely taught in medical schools, despite the growing challenges faced by healthcare institutions.
**Knowledge Generated**
We applied a validated leadership competencies instrument to a sample of 217 Latin-American physicians-leaders from oncology and related areas.
**Relevance**
We documented their unique perceptions towards leadership learning and real-life applications.

In 2009, Violato and Cawthorpe^14^ identified key competencies for scholars, teachers, researchers, and leaders in medical education. This study proposed a framework for the development of a model for evaluation of LCs in medical education (and possibly in the health care sector in general). Çitaku et al^15^ further developed this concept and performed a population-based survey aiming to identify perceived competencies for medical leadership in medical education in a relatively multicultural context (although restricted to Western and developed countries). The 229 participants were educators, physicians, nurses, and other health professionals who held academic positions in medical education. The authors adapted a 107-item LC scale developed by Wagner et al^16^ into a 63-item questionnaire to be more relevant to the field of medical education. In contrast with the results of Wagner et al,^16^ Çitaku et al^15^ identified social responsibility as the most important competency, which was attributed to an emphasis on collaboration and interdisciplinary practice in medical education (as opposed to competition and individualism in the business setting). Although this 63-item questionnaire represents a potential tool for training physicians in leadership skills, it should be studied for its cross-cultural validity in other cultures in the world.

The objectives of our study were as follows: to offer this survey to a population of LA physicians from the oncology community and related areas who hold leadership positions of various levels; to compare these results with those of the previous survey; and to investigate potential interactions between LA physicians’ perceptions of LCs and factors such as specialty, country of medical practice, sex, type of medical practice (private *v* public), age, years of experience in oncology, and years of experience in leadership position.

## METHODS

### Participants

The 63-item questionnaire was sent to physicians who were full members of an LA cooperative cancer research group or of one of the medical societies that agreed to participate. All institutions were in some way involved with cancer care, although for 2 of them (the mastology and pathology societies), oncology was not an exclusive activity. The characteristics of these institutions are listed in Table 1. The survey had a standard introductory message that explained the goal of the study and clarified that the physician should only proceed to completing the questionnaire if he or she had an ongoing leadership position.

**TABLE 1:**
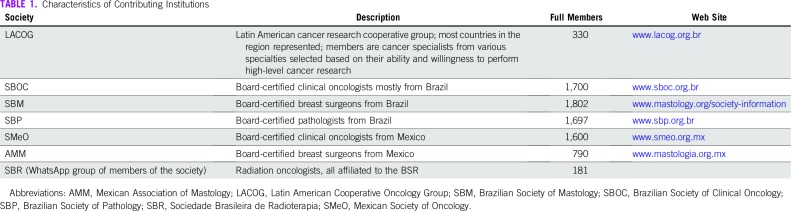
Characteristics of Contributing Institutions

### Procedures

As a result of internal compliance issues, the mechanism of survey distribution differed for each medical society. The Latin American Cooperative Oncology Group (LACOG) was the primary sponsor of the study and was able to provide the membership mailing list, which enabled us to send the survey from the first author’s e-mail through SurveyMonkey (San Mateo, CA), allowing us to send reminders (up to a maximum of 4). For all other organizations, the e-mail message had to be sent via institutional official e-mails. For the Brazilian Society of Medical Oncology, the Mexican Association of Mastology, and the Brazilian Society of Pathology, the invitation was sent only once. For the Brazilian Society of Mastology (SBM) and the Mexican Society of Oncology, the invitation was sent twice. The Brazilian Society of Radiation Oncology (SBR), because of its strict antispam policies, was not able to officially participate or respond to the survey; however, the physicians held an active WhatsApp (Menlo Park, CA) group in which the majority of radiation oncologists with leadership positions were represented, so we decided to circulate the survey through this network. In this case, we sent 3 reminders in total.

In line with methods previously used by Çitaku et al,^15^ we grouped the questions into 5 major categories (task management, self-management, social responsibility, innovation, and leading others). The study was waived formal submission to the ethics committee to which LACOG reports.

### Analyses

For the purpose of comparisons, we analyzed the results in terms of proportion of participants who responded with a 4 or 5 (ie, “this competence is important” or “this competence is very important,” respectively) on the survey. Survey respondents’ characteristics and the responses were summarized using descriptive statistics. Between-group differences were analyzed using contingency tables (χ^2^ test). Internal consistency reliability was computed (Cronbach’s α = .830294). All analyses were performed using the SAS statistical software (version 9.4; SAS Institute, Cary, NC). A significance level of 5% was applied.

## RESULTS

From November 13, 2018, to December 12, 2018, we sent the electronic invitations to almost 8,000 physicians and collected a total of 217 responses. Because the responses were anonymous, we were not able to track the response rate for most of the societies. However, considering the size of each society, the return rate is assumed to have been < 10%. For SBM, for instance, the survey was sent to 1,803 members, and 92 clicked to fill out the survey, yielding a response rate of 5.1%. For the SBR, we obtained a total of 14 responses from 181 participants of the WhatsApp group, yielding a response rate of 7.73%. We sent the survey to 330 LACOG members and collected 74 responses, yielding a more robust response rate of 22.42%.

The majority of the scores were 4 or 5 (ie, the competence is important or very important, respectively), but for most of the questions, responses were distributed from 1 to 5. In addition to the 63 questions with 5 possible responses, we also collected data on type of institution (private *v* public), country of medical practice (Brazil, Argentina, Uruguay, Peru, Chile, Paraguay, Colombia, Mexico, or other), main specialty (clinical oncology, radiation oncology, surgical oncology, palliative care, or other), sex (male, female, or other/prefer not to inform), age, years of experience in oncology, and years of experience in a leadership position. The characteristics of the research participants are listed in Table 2.

**TABLE 2:**
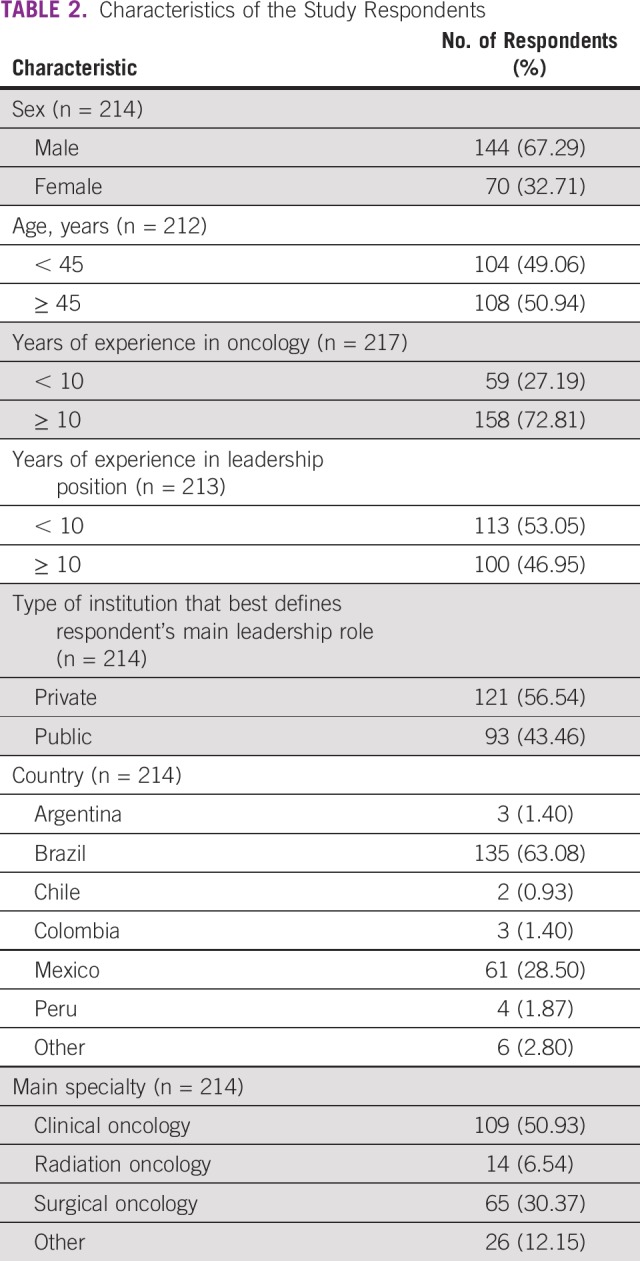
Characteristics of the Study Respondents

LA physician-leaders placed the highest value on task management competencies, with a rate of responding with a 4 or 5 of 91.37%. This was statistically significantly higher than rate for North American (NA) and European Union (EU) participants (87.0%; *P* < .0001). The second highest value was placed on self-management competencies (87.45%), followed by social responsibility competencies (86.83%). The second lowest rate was found for innovation competencies (86.69%), and the lowest for leading others competencies (83.31%). Except for task management, no other significant differences between the LA and NA/US surveys were observed. These results are listed in Table 3.

**TABLE 3:**
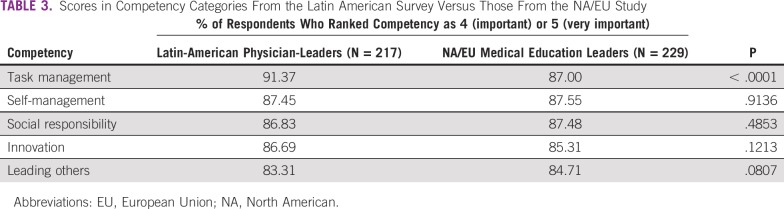
Scores in Competency Categories From the Latin American Survey Versus Those From the NA/EU Study

We performed subgroup analyses, the results of which are listed in Table 4. In the comparison of public versus private institution, we found no significant differences; there was only a trend toward physician-leaders from public institutions placing a higher value on social responsibility competencies compared with leaders from private services (88.31% *v* 84.1%, respectively; *P* = .0675). In the country comparisons, participants from Brazil, compared with participants from other LA countries, gave statistically significant higher scores for the competencies of task management (93.32% *v* 88.04%, respectively; *P* = .0004), social responsibility (88.73% *v* 84.16%, respectively; *P* = .0001), self-management (88.71% *v* 85.1%, respectively; *P* = .0108), and leading others (84.53% *v* 81.23%, respectively; *P* = .0064). In the medical specialty comparison, clinical oncologists, compared with other specialties, placed a higher value on social responsibility (89.55% *v* 83.87%, respectively; *P* < .0001) and a lower value on leading others (82.03% *v* 84.64%, respectively; *P* = .0243) and innovation (85.18% *v* 88.23%, respectively; *P* = .0146). In the sex comparison, women placed a higher value on innovation competencies compared with men (89.32% *v* 85.44%, respectively; *P* = .004). In the age analysis, leaders > 44 years old, compared with younger leaders, provided higher scores on task management (93.38% *v* 89.13%, respectively; *P* = .0038) and leading others (85.03% *v* 82.01%, respectively; *P* = .0098). Leaders with 10 or more years of experience in oncology, compared with those with < 10 years of experience, provided higher scores for the competencies of task management (92.84% *v* 87.24%, respectively; *P* = .0007) and leading others (84.69% *v* 79.42%, respectively; *P* = .0001). Leaders with 10 or more years of experience in a leadership position, compared with less experienced medical leaders, also rated higher scores on the competencies of task management (93.56% *v* 89.75%, respectively; *P* = .0083) and leading others (85.25% *v* 81.81%, respectively; *P* = .0033).

**TABLE 4:**
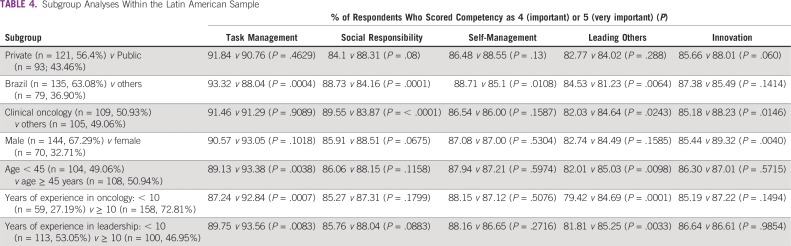
Subgroup Analyses Within the Latin American Sample

## DISCUSSION

To our knowledge, this is the first study to comprehensively address the perception of LA physicians on the relative importance of a number of LCs and one of the largest studies ever performed specifically with physicians. We contacted nearly 8,000 physicians from the oncology community and related areas to collect a total 217 responses, which provides statistical power for the analyses.

Our data show that LA medical leaders place the highest value on task management competencies. Furthermore, the results registered for task management by LA medical leaders were statistically significantly higher than those of NA/EU medical education leaders (91.37% *v* 87.0%, respectively; *P* < .0001), for whom task management competencies were only third in importance.^15^ The reasons for this difference are not obvious but could reflect cultural differences in the importance of completing tasks. However, for the other 4 clusters of LCs, we found no significant differences between LA and NA/EU scores.

Of note, social responsibility, which was of highest importance in the previous NA/EU survey,^15^ only ranked third in importance in the LA survey. This difference could be a result of the higher representation of physicians in the current survey (100% in the current survey *v* 39.63% in the NA/EU survey) because, in the NA/EU survey, physicians tended to place a lower value on social responsibility competencies (which was assumed to be attributable to the deeper human involvement and sense of responsibility of nonmedical staff toward patients, families, and the community). These differences between physician and nonphysician aspiring leaders have also been noted in other studies, especially in terms of personality traits.^17^

Our findings of significant differences in the valuation of LCs in an LA population compared with other world cultures are somewhat consistent with data reported in other domains. For instance, in the GLOBE survey, respondents from LA placed a higher value on in-group collectivism and a lower value on performance orientation, future orientation, institutional collectivism, and uncertainty avoidance. They also valued loyalty and devotion to the family and other groups but, at the same time, were less attentive to group issues in the workplace. When questioned about desired leadership characteristics, they favored a charismatic and value-based, team-oriented, and self-protective leadership style and were somewhat averse to an autonomous leadership style. However, we cannot directly compare the nature of our results with those of the GLOBE study because the latter was performed in a different domain and also used different evaluation criteria.^12^

In the private versus public comparison, there were some trends that did not reach statistical significance. This might have occurred because in LA it is common for physicians to hold both private practice and public or academic positions concurrently, so they might represent a similar population. As a result, the question was framed in terms of “describe the type of institution that best defines your main leadership role”; otherwise, it would generate ambiguity.

There were a number of differences for other subgroup analyses. For instance, clinical oncologists placed a statistically significantly higher value on social responsibility competencies compared with other specialists. This might be a result of their deeper level of emotional involvement with patients and their families and their full understanding of the devastating impact of cancer on the patients, families, and the whole community.^18,19^ This could also explain the trend for a higher valuation of social responsibility competencies by medical leaders from public services compared with leaders from private services, with the former group assisting a more vulnerable patient population^20^ and, as such, being more connected with the nonnegligible social burden of this disease.^21,22^

One of the most striking findings of our study was the fact that more senior leaders, as measured by age (≥ 45 years), years of experience in oncology (≥ 10 years), or years of experience in a leadership position (≥ 10 years), compared with less experienced respondents, consistently placed greater value on the competencies of task management (93.38% *v* 89.13%; *P* = .0038; 92.84% *v* 87.24%; *P* = .0007; and 93.56% *v* 89.75%; *P* = .0083, respectively) and leading others (85.03% *v* 82.01%; *P* = .0098; 84.69% *v* 79.42%; *P* = .0001; and 85.25% *v* 81.81%; *P* = .0033, respectively). Task management and leading others represent a set of complex and critically important skills that are only fully understood and acquired with seniority.

In contrast to the study by Çitaku et al,^15^ which included a disproportionately high number of women (60.9%), our survey included a majority of men (67.3%). This is another interesting finding that could be explained by an underrepresentation of women in medical leadership positions in the region. Conversely, in the study by Çitaku et al,^15^ > 60% of respondents were nonphysicians, a group that could have a disproportionate number of women professionals. Interestingly, in our study, women placed a statistically significant higher value on innovation competencies compared with men (89.32% *v* 85.44%, respectively; *P* = .0040). This may reflect true cultural differences between male and female medical leaders. Of note, studies performed mainly in the business realm indicate the existence of modest differences in leadership styles, with women more often using democratic and transformational leadership^23^ and being more entrepreneurial than men.^24,25^ Furthermore, female doctors seem to be no less interested than men in developing LCs, as demonstrated by a high attendance rate of 72% in a leadership training program for hospital-based physicians.^7^ However, recent data suggest that women remain underrepresented in leadership positions in many areas of medicine.^26,27^

There are other findings from the subgroup analyses that may also reflect true cultural differences between the subgroups of participants. For instance, other specialists, compared with clinical oncologists, tended to place a higher value on the competencies of leading others (84.64% *v* 82.03%, respectively; *P* = .0243) and innovation (88.23% *v* 85.18%, respectively; *P* = .0146). It may be that, in a sample mainly composed of radiation and surgical oncologists (ie, other specialists), innovation is deeply rooted in the complex and rapidly evolving technologic aspects of their specialties, in contrast with clinical oncologists, who spend long hours in deep talks and reflections with their patients. Finally, Brazilian physicians placed a statistically significant higher value on the competencies of task management, social responsibility, self-management, and leading others than other LA physicians. This may be because of the many analyses we conducted resulting in a type I error or perhaps a sampling artifact.

Even though we applied the same questions used in the NA/EU survey, grouping the 63 individual competencies into 5 major sets of LCs according to their meaning, Çitaku et al^15^ followed a slightly different track by applying specific statistical tests to come up with another domain, which was named justice orientation competencies. We used a different set of statistical tools and retained the original proposal of 5 major domains; thus, we kept innovation competencies and did not include justice orientation competencies, which is closer to the original survey by Wagner et al^16^ from which the current questionnaire was derived.

The low response rate for the majority of the societies is worth noting. However, we also understand that this is compatible with the scope of the questionnaire; although LACOG membership likely consists of physicians with leadership roles (thus yielding a higher response rate of 22.42%), only a small fraction of members of specialty medical societies are expected to hold active leadership positions. The higher return rate for LACOG members could also be a result of the more personal nature of the invitation to participate and the ability to send reminders to this group. In short, this implies that, despite the fact that holding a leadership role was self-defined in this study, we might have succeeded in reaching the target population.

Strengths of our study include the following: the sample size was large; there was rich cultural diversity of the respondents; it targeted physicians specifically, contributing to the homogeneity of the study population; the research was supported by major multinational collaborative research groups and various medical societies; and it applied rigorous methods in terms of unbiased participant selection.

We believe there are 6 limitations of our study. They are as follows: the response rate was generally low; holding a leadership position was self-defined; there is a perceived risk that major leaders are so busy that they were unable to complete or did not even receive the survey; the survey was not translated to the physicians’ native languages (which could be detrimental because the survey deals with relatively abstract concepts); the respondents were mostly in some way connected with the oncology community (although not exclusively and with various different specialties having contributed to the responses); and we cannot guarantee that all LA respondents were involved with medical education (which was a selection criterion in the study by Çitaku et al^15^).

By successfully applying a validated survey to 217 LA medical leaders from the oncology community and related areas, we identified significant cultural variations in the valuation of a set of specific medical LCs. These differences were evident for 1 specific domain (task management competencies) when we compared LA with NA/EU responses, but not for the other 4 domains.

We also performed subgroup analyses (country of medical practice, age, years of experience in oncology, years of experience in a leadership position, sex, specialty, and type of medical practice), which revealed significant interactions between these factors and the valuation of specific LCs within the LA sample of medical leaders.

Considering the pressing need to develop leadership training programs for current and aspiring medical leaders, our data will hopefully help to shape the content of these programs to meet unique regional needs. Finally, given the importance of the subject, further studies should attempt to apply this same questionnaire in other clusters of world culture.
